# From first encounters to longitudinal exposure: a repeated exposure-test paradigm for monitoring speech adaptation

**DOI:** 10.3389/fpsyg.2024.1383904

**Published:** 2024-05-30

**Authors:** Xin Xie, Chigusa Kurumada

**Affiliations:** ^1^Department of Language Science, University of California, Irvine, Irvine, CA, United States; ^2^Department of Brain and Cognitive Sciences, University of Rochester, Rochester, NY, United States

**Keywords:** nonnative accent, adaptive speech perception, repeated exposure-tests, longitudinal testing, online perceptual experiment

## Abstract

Perceptual difficulty with an unfamiliar accent can dissipate within short time scales (e.g., within minutes), reflecting rapid adaptation effects. At the same time, long-term familiarity with an accent is also known to yield stable perceptual benefits. However, whether the long-term effects reflect sustained, cumulative progression from shorter-term adaptation remains unknown. To fill this gap, we developed a web-based, repeated exposure-test paradigm. In this paradigm, short test blocks alternate with exposure blocks, and this exposure-test sequence is repeated multiple times. This design allows for the testing of adaptive speech perception both (a) within the first moments of encountering an unfamiliar accent and (b) over longer time scales such as days and weeks. In addition, we used a Bayesian ideal observer approach to select natural speech stimuli that increase the statistical power to detect adaptation. The current report presents results from a first application of this paradigm, investigating changes in the recognition accuracy of Mandarin-accented speech by native English listeners over five sessions spanning 3 weeks. We found that the recognition of an accent feature (a syllable-final /d/, as in *feed*, sounding/t/-like) improved steadily over the three-week period. Unexpectedly, however, the improvement was seen with or without exposure to the accent. We discuss possible reasons for this result and implications for conducting future longitudinal studies with repeated exposure and testing.

## 1 Introduction

How listeners navigate the substantial amount of cross-talker variability is a central question in speech perception. The “same” phonological category or word is produced with distinct acoustic-phonetic properties across talkers with different characteristics (e.g., height, gender, accent). This variability is known to make the recognition of unfamiliar talkers or accents difficult (Adank and Janse, 2010; Porretta et al., 2016). These difficulties can, however, dissipate as listeners adapt to the current input (Bradlow and Bent, 2008; Tzeng et al., 2016; Baese-Berk et al., 2020; Xie et al., 2021). For example, native listeners of English become significantly faster and more accurate in responding to Spanish-or Mandarin-accented speech within as few as 18 sentence-length utterances (Clarke and Garrett, 2004; Xie et al., 2018).

Among the speech variants used to study adaptive perception, nonnative accents have several unique properties. Most prominent are the complex ways in which they deviate from the native variants, both at the segmental and suprasegmental levels. Unlike other types of acoustically degraded or noisy speech, accented speech is difficult to understand primarily because it alters how acoustic cues map onto speech categories such as phonemes and words. In some cases, one category is phonetically confusable with another (e.g., a voiced stop consonant like the [d] in “seed” is often devoiced in a word-final position that sounds more like the [t] in “seat” in German and Dutch accented English, Eisner et al., 2013); in others, categories are merged, substituted, or omitted (e.g., the English /θ/ is substituted by different categories across accents Hanulíková and Weber, 2012, for a review see Bent and Baese-Berk, 2021). These variations can lead to lexical ambiguity and uncertainty, often resulting in slower and less accurate recognition.

While these variations may be idiosyncratic, they are far from random. Talkers from similar native language (L1) backgrounds tend to share common accent features, influenced by L1 phonology and its difference from the nonnative (L2) phonology (Flege et al., 1992; Munro and Derwing, 1995). Critically, L1 effects are highly category-and cue-specific, creating a “learnable” statistical structure (Vaughn et al., 2019; Xie and Jaeger, 2020). Indeed, Eisner et al. (2013) demonstrated that British English listeners adapted to the word-final devoicing in Dutch-accented English, a finding that has since been extended to other L1-L2 accents (e.g., Mandarin-accented American English, Xie et al., 2017). Rapid adaptation has been seen in populations with varying auditory sensitivity and memory capacity (Bieber and Gordon-Salant, 2017, 2021) and can generalize (albeit with limits) across talkers who share an accent (Baese-Berk et al., 2013). Exposure benefits in nonnative accent adaptation have thus served as a rich testbed for theories of perceptual learning, adaptation, and its generalization.

Beyond relatively short-term adaptation, real-world speech recognition tends to evolve over repeated episodes of social interaction across talkers and contexts distributed over much longer time spans. Long-term familiarization with an accent over months and years can facilitate the comprehension of, and adaptation to, a novel talker from the same or similar accent background (Weber et al., 2014). Witteman et al. (2013b) tested native Dutch listeners with limited or extensive prior experience with German-accented Dutch in spoken word recognition. Only those with extended experience with the accent were able to activate the correct lexical entities when hearing heavily accented tokens. Porretta et al. (2016) further demonstrated a gradient effect of accent familiarity on the lexical processing of spoken words. From these experimental results, and many personal anecdotes, it is tempting to conclude that repeated exposure accumulates to support adaptive speech perception. However, it is also known that environmental exposure to a previously unfamiliar accent alone does not always lead to a significant change of perception (Evans and Iverson, 2007).

Thus, it remains an open question how much exposure could lead to stable, long-lasting perceptual benefits, and existing results are mixed. Witteman et al. (2015) showed that adaptation induced by only 3.5 min of exposure could be detected as far out as a week later. On the other hand, Bieber and Gordon-Salant (2017) found that neither younger nor older adults retained the initial benefit in a delayed test 7–10 days after exposure (see also Zheng and Samuel, 2023). These could be due to differences in methods (e.g., cross-modal priming vs. speech repetition), accent types (e.g., Hebrew-accented Dutch vs. Spanish-accented English), or measures (e.g., adaptation to a single talker vs. generalization across talkers and accents). Regardless, an important gap in the knowledge is whether and if so, how short-term adaptation relates to more long-term changes of perception and/or learning of underlying linguistic representations (for reviews, see Bieber et al., 2023; Xie et al., 2023).

While there is a growing interest in adaptation across various timescales (Bieber and Gordon-Salant, 2017; Banai et al., 2022; Bieber et al., 2023; Zheng and Samuel, 2023), empirical investigation into the medium-term effects—spanning days to weeks—remains limited. Furthermore, most existing data are from a single, delayed test. They therefore provide little information about the effects of repeated exposure to the same accent, although such interactions are common in real-life social, educational, and workplace settings. Would listeners maintain adaptive changes across these encounters, or would they start over each time? This gap underscores the need for mid-to long-term study designs that more accurately reflect everyday accent exposure and adaptation processes.

Two major challenges remain. The first is subject retention, in particular, to scale up the paradigm to even longer time periods with more frequent tests than the ones considered here. We approached this challenge by using a web-based paradigm that has previously been employed in single-session experiments. Participants are recruited from an online research participant recruitment platform (e.g., Prolific). Making the experiment fully online substantially lowered the effort required by participants (e.g., no need to visit the lab), increasing accessibility and achieving manageable subject attrition (<30% over 3 weeks). An equivalent in-lab procedure would be more time-consuming for both participants and researchers, which would limit experimental design options, subject eligibility, and retention.

A second challenge—one of relevance to research on adaptive speech perception in general—is that any test also constitutes a form of exposure, so repeated testing can interfere with researchers’ ability to accurately measure the effects of exposure. Consider a scenario in which one group is exposed to L2-accented speech, and the other to L1-accented speech. Repeated testing on L2-accented speech tokens inevitably dilutes the difference between the two groups. In other words, the test tokens themselves provide participants with information about the target accent, even in the absence of audio-visual, lexical, or other context that effectively labels the input (Maye et al., 2002; Clayards et al., 2008). Test stimuli that are often thought of as “neutral,” such as those sampled uniformly across a continuum, are not free of bias. Listeners can learn the unique statistic in the test stimuli, which gets integrated into and eventually overrides exposure effects. In fact, recent papers provide evidence that prolonged testing reversed adaptive changes that occurred during exposure (Liu and Jaeger, 2018; Cummings and Theodore, 2023; Zheng and Samuel, 2023).

**Figure 1:**
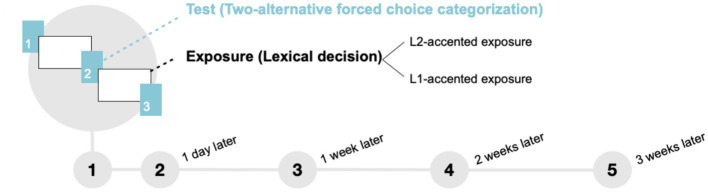
Each session consisted of three test blocks (10 items each) interspersed with two exposure blocks (90 items each) within a single session. This block design was repeated five times: Day 1, 1 day later, 1 week later, 2 weeks later, and 3 weeks later.

To address these challenges, we developed a new testing paradigm that balances two competing motivations. On the one hand, we need to keep the number of trials to a minimum to not interfere with exposure. On the other hand, we need to ensure sufficient statistical power to detect the effect of exposure. To achieve this, we created a repeated-exposure-test protocol in which three short (10-item) test blocks alternate with two relatively long exposure blocks within a single session (Figure 1). We then repeat the five-block design five times. This allows us to keep each test block short, while increasing the total number of test trials and statistical power of the data. In addition, this new protocol enables us to accomplish the overarching goal of tracking the development of adaptation on multiple time scales, from after the first few minutes of exposure to up to 3 weeks.

Our design built on Xie et al. (2017), who used a single-session exposure-test design to examine L1-English listeners’ adaptation to a Mandarin-accent word-final /d/-/t/ contrast in English. A syllable-final /d/ vs. /t/ in this accent is often contrasted by burst duration, rather than the cues expected to be most informative in L1-accened English (closure and vowel duration, for more details see 2.2). Many instances of a syllable final /d/ (e.g., “kid”) sound like a /t/ (e.g., “kit”) to L1 listeners, and adaptation includes learning to upweight the burst duration over the other cues. That is, rather than examining global improvements independent of accent features, this study zoomed in on how L1 listeners learn new acoustic cue distributions for this specific contrast through exposure to natural Mandarin accents.

Xie et al. (2017) used a between-subjects manipulation in which native listeners of American English answered 180 lexical decision questions in the exposure phase. Participants in the target condition heard 30 (11%) items containing a syllable-final /d/ sound in a lexically-biased context (e.g., “lemonade”), which was expected to support their adaptation to the accent feature. In the control condition, these items were replaced with words without a syllable-final /d/ sound, and no adaptation was expected. No other stop sounds were present at syllable-final position throughout exposure. During the test, all participants responded to 60 minimal pairs (i.e., 120 items) in a phonetic categorization task (e.g., “kid” or “kit”?). They found that exposure to the critical accent feature significantly improved categorization accuracy of /d/, reflecting adaptation to the nonnative accent feature.

As in Xie et al. (2017), one group of participants in the current experiment were exposed to Mandarin-accented US English, where the critical words contain a syllable-final /d/ sound. Unlike in Xie et al. (2017), the control participants heard an L1-accented (i.e., native) US English talker producing the same set of words, including the critical words with a syllable-final /d/ sound. Although none of the original exposure items contained stop voicing contrasts, there may be other accent features covarying with the relevant /d/-/t/ contrast (e.g., the realization of voicing in fricatives) which may aid adaptation. This concern is particularly strong for our current multi-session protocol, where a listener receives a large number of exposure trials to a particular talker (180 * five sessions = 900 trials) over five sessions. We therefore used L1-accented talker in the control condition to remove this possible confound.

During test, both groups were tested on the same set of Mandarin-accented L2 US English tokens which did not occur during exposure. Each test block was brief (10 items from five minimal pairs), and the same set of items were repeated in all test blocks. To counteract the reduced number of test items per block [five (8.3%) out of 60 pairs from Xie et al., 2017], we carefully selected stimuli that were predicted to increase statistical power of the results (see details in the Methods section).

We considered two broad classes of results, each of which could shed light on how adaptation develops over days and weeks. If adaptation occurs rapidly but also decays rapidly, benefits originating from the L2-accented (vs. L1-accented) exposure should be found within each session (Eisner et al., 2013; Xie et al., 2017) but not accumulate across sessions. On the other hand, if immediate and rapid adaptation does lead to enduring changes, the exposure benefits should interact with the number of blocks, e.g., the accuracy difference between the L2-accented vs. L1-accented exposure conditions should increase over the 15 test blocks. Due to the novelty of the paradigm, some of the methodological decisions were made based on related studies. Much of the empirical data needed to formally test hypotheses were not available (e.g., effect sizes and participant attrition rates across multiple sessions). The current methods are thus meant as our initial attempt. In General Discussion, we suggest potential refinements based on the data from this study.

## 2 Methods

All data, analysis scripts, and model summaries are downloadable from OSF (osf.io/5xfpe/).

### 2.1 Participants

An initial group of 127 monolingual, native speakers of American English, aged 18–45, were recruited via Prolific^1^ and completed Session 1 of the experiment via the online testing platform FindingFive.^2^ Because the precise estimates of effect sizes and participant attrition rates were *a priori* unknown, the initial recruitment goal was set based on the published work (Xie et al., 2017). The original work included 24 participants in each condition (48 in total); this sample size was equal to or larger than that in other similar work investigating accent adaptation (e.g., Eisner et al., 2013; Witteman et al., 2013a; Zheng and Samuel, 2020). To buffer against subject attrition and increased response variability expected in online testing, we recruited 60–65 participants (i.e., approximately 250% increase) in each condition in Session 1.

Due to an administrative error after Session 1, which has subsequently been corrected, 30 participants were unable to continue to the following sessions (see Supplementary material). Of the remaining 97 participants, 70 participants (71%) completed all five sessions (*n* = 32 in the L2-accented exposure condition; n = 38 in the L1-accented exposure condition). Thus, the attrition rate for the last four sessions spanning 3 weeks was 27.8%, and comparable between the two exposure conditions: ~28.3% for the L2-accented exposure (15 out of 53) and ~ 27.2% for the L1-accented exposure (12 out of 44).

The 70 participants included in the analysis were recruited from 38 US states, and self-identified as native speakers of US English. Only 6% (four out of 70) of participants reported that they regularly hear Mandarin Chinese spoken by a family member or a close friend; three of them also reported having parents who speak English with a nonnative accent. As we reported in the Supplementary material, excluding these four participants did not change the results.

### 2.2 Stimuli

*Exposure stimuli* for the L2-accented exposure group consisted of 90 English words (30 critical and 60 filler items) and 90 phonotactically-legal nonwords. The critical items were all multisyllabic words ending in /d/ (e.g., lemonade, overload). The exposure list for the L1-accented exposure group was identical except that they were produced by a native speaker of American English. Filler words and nonwords did not contain any /d/ or /t/ sounds, and no stop sounds other than /d/ appeared in the word-final position. The exposure items were evenly distributed across the two exposure blocks, each of which thus contained 50% of the exposure stimuli from Xie et al. (2017). The word-block assignment was counterbalanced across participants and remained constant within participants across the five sessions. Item presentation was randomized within each block.

*Test stimuli* consisted of five /d/-/t/-final minimal pairs (e.g., *feed-feet*; 8% of Xie et al., 2017), constituting 10 trials per block. This small set of test items was intended to minimize the interference with exposure effects. To increase the chance of detecting adaptation, we selected test tokens that were predicted to be categorized differently after L1-and L2-accented exposure (e.g., a /d/-ending word incorrectly recognized as *_t* after L1-accented exposure but correctly recognized as *_d* after L2-accented exposure). To the extent that past work has taken similar steps, this has typically been focused on the selection of test *talkers* rather than the selection of specific *stimuli*. For example, it is common to select L2 talkers with low-to-medium intelligibility to avoid floor and ceiling effects. However, the effectiveness of an individual stimulus token is known to vary *within* a talker, depending on their exact acoustic-phonetic properties (Burchill, 2023; Xie et al., 2023).

**Figure 2:**
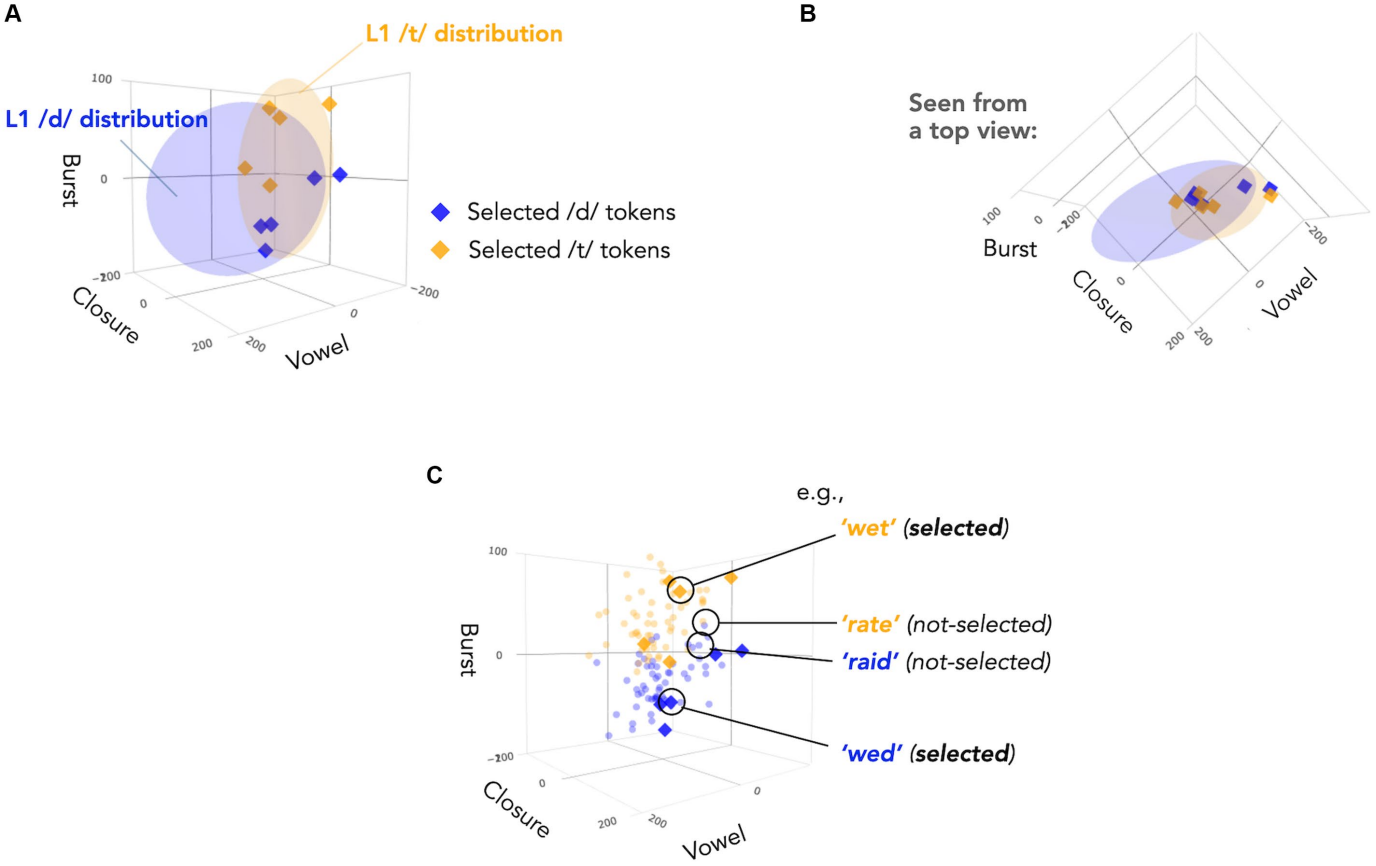
**(A)** The selected test items (diamonds) plotted against L1-accented /d/ and /t/ categories in a three-dimensional talker-normalized phonetic space (vowel, closure and burst; for details, see Tan et al., 2021). The ellipses show 95% probability density of multivariate Gaussian categories. For details of data used to estimate the distributions, see Tan et al. (2021). **(B)** Same as Panel **(A)**, but seen from a top view, emphasizing the distribution along closure and vowel duration. The selected test items fall into an ‘ambiguous’ region of the acoustic-phonetic space where L1 /d/ and /t/ overlapped. **(C)** The selected test items (diamonds) plotted against the other 50 test item pairs used in Xie et al. (2017) (circles). While burst duration is generally informative about a given item’s category membership (=/d/ or /t/?), the informativeness varies across items. The selected pairs were those predicted by models of distributional learning to yield major improvements in the recognition accuracy after the L2-accented exposure (relative to L1-accented exposure). A contrast between a selected pair (*wet* vs. *wed*) and a pair not selected (*rate* vs. *raid*) is highlighted to illustrate this point.

With this in mind, we first examined the acoustic cues of all 60 pairs of test items used in Xie et al. (2017) across the three cue dimensions: burst, closure, and vowel. We compared them to typical distributions in L1-accented speech, illustrated by blue and yellow ellipses in Figure 2A. As noted above, the L1 category distributions are primarily separated by closure and vowel duration; in contrast, L2 Mandarin-accented talkers tend to use burst duration, leading to an overlap in the other two dimensions between /d/ and /t/ categories (Figure 2B) and potential confusion for L1 listeners. While L1 listeners may theoretically resolve this confusion by placing more the perceptual weight on burst duration as a cue for distinguishing /d/ and /t/ sounds, the informativeness of burst duration varies across items due to interactions with the other two cues (Figure 2C).

We then used a model to predict how listeners would respond to *each* test token under different exposure conditions, considering all three acoustic cues (vowel, closure, and burst duration). Tan et al. (2021) used Bayesian ideal observer models to simulate the outcomes of the two exposure conditions from Xie et al. (2017). Each model’s /d/ category was trained on the respective exposure tokens annotated for the three cues. Since exposure in both groups never included instances of /t/, the /t/ category for both exposure conditions were trained on US English /t/-productions, based on the assumption that L1 listeners in both groups would apply their *a priori* (= L1-based) expectation for the /t/ category. Their results showed that model predictions significantly predicted human categorization responses in each exposure condition at the token level.

**Figure 3:**
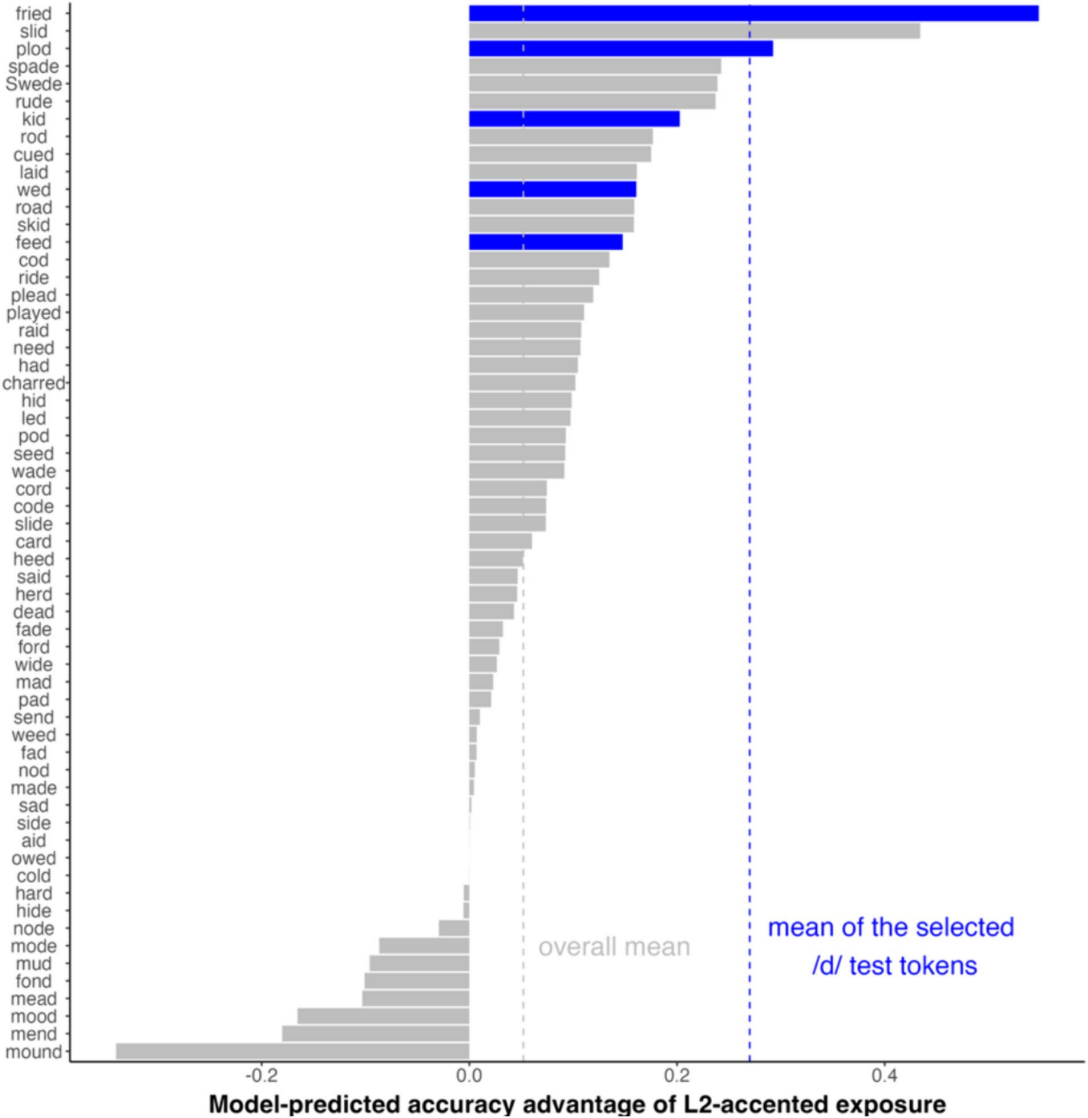
/d/ test tokens ranked by the model-predicted accuracy advantage of L2-accented over L1-accented exposure conditions. The tokens selected for the current experiment are highlighted in blue. Vertical dashed lines indicate the average for all the 60 pairs from Xie et al. (2017) (gray) and the five selected pairs (blue).

We applied the same simulation approach, using MVBeliefUpdatr (Jaeger and Burchill, 2021) along with the R code distributed as part of Xie et al. (2023). We ranked all the 60 pairs of test items in terms of the predicted L2-accent exposure advantage (Figure 3). From the ranked items, we selected five pairs associated with a strong advantage while controlling other factors that would plausibly affect the effectiveness of the test items (e.g., word frequency, vowel types, and ceiling/floor effects on both the /d/ and the /t/ members of a minimal pair). The selected items were thus associated with a significantly higher level of L2-accent exposure advantage, well above the mean of the original 60 pairs.

### 2.3 Procedure

Five experimental sessions were administered on 5 days over the course of 3 weeks (Figure 1). Each session consisted of three test blocks (10 trials each) interleaved with two exposure blocks (90 trials each). After a headphone check to adjust volume and confirm the audibility of the audio stimuli, participants began with a test block. Participants were informed that during this block they would hear words ending in /d/ or /t/ and asked to provide two-alternative forced choice (2AFC) responses to the question “Did you hear a D or a T?” The 10 items from five minimal pairs (e.g., “kid” or “kit”) were presented in random order without repetition within each test block.

During exposure, participants completed a lexical decision task (i.e., word or nonword). 180 trials from Xie et al. (2017) were equally divided between two lists presented in the two exposure blocks. Participants heard one token at a time and responded whether it was a real word of English (e.g., “lemonade”) or a nonword (e.g., “salvary”). After the five test/exposure blocks, all participants completed a questionnaire about their language background and familiarity with L2 accents in the first session (Day 1). The median participation time was 23 min per session.

Using Prolific’s “custom allow list” feature, we invited the participants back to our experiment four more times. We also used Prolific’s communication system to send periodic reminders to reduce attrition. During each of Sessions 2–5, the experiment was open for 24 h starting at 9 am Pacific time on a given day, ensuring that the interval between sessions was 1 day (sessions 1–2) and 1 week (after session 2), while the exact interval duration varied across participants. Delayed participation beyond this 24 h window was not permitted. We note that all but three participants across all five sessions completed the experiment between 9 am-10 pm Pacific time, with the majority completing the experiment in the morning and afternoon before 6 pm.

## 3 Results

### 3.1 Exposure

**Figure 4:**
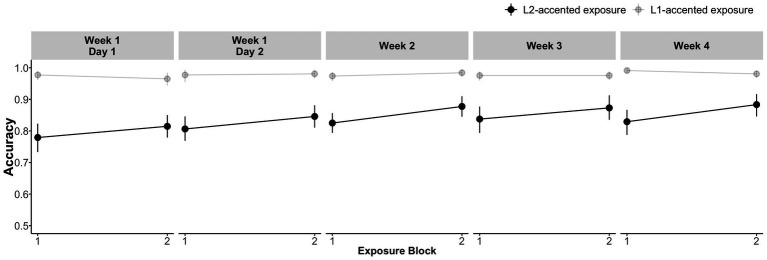
Recognition accuracy for the critical /d/-final words in the auditory lexical decision task during exposure blocks spanning 3 weeks. Error bars represent bootstrapped 95% confidence intervals over by-participant means.

Figure 4 shows the overall performance on the lexical decision task during exposure. As expected, participants in the L2-accented exposure group had lower accuracy (mean = 0.85; SD = 0.03) than the L1-accented exposure group (mean = 0.96; SD = 0.03). Focusing on the critical /d/-final words, the L2-accented exposure group showed a steady improvement within each session and across sessions (1^st^ block; mean = 0.78, SD = 0.12; last block: mean = 0.88, SD = 0.11). Meanwhile, performance in the L1-accented exposure group was near ceiling throughout (1^st^ block; mean = 0.98, SD = 0.04; last block: mean = 0.98, SD = 0.04). The incremental improvement in the L2-accented exposure group suggests that (1) even 15 critical items per exposure block were sufficient to enhance recognition of L2-accented speech, and (2) these enhancements accumulated with increasing exposure.

### 3.2 Test

**Figure 5:**
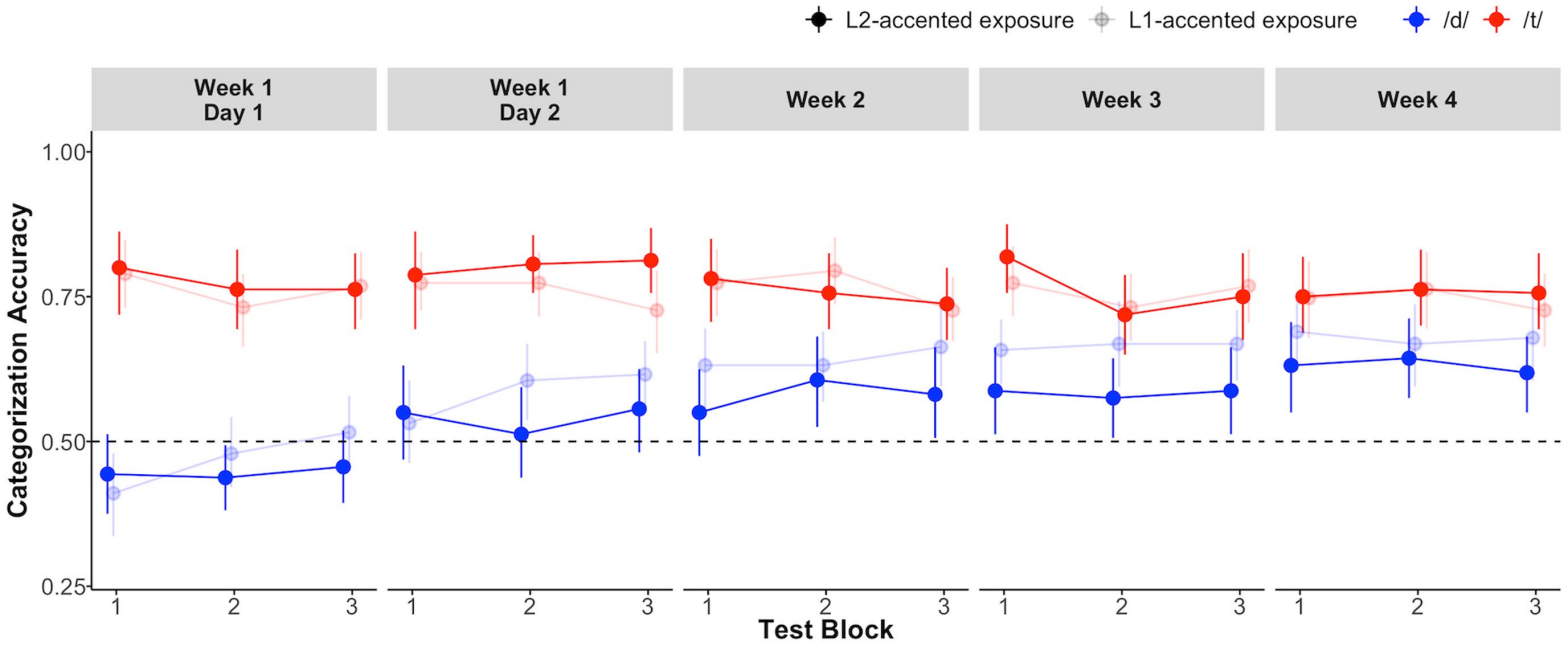
Performance during the test blocks spanning 3 weeks. Error bars represent bootstrapped 95% confidence intervals over by-participant means.

Figure 5 summarizes participants’ categorization accuracy on the L2-accented test tokens. As predicted, recognition of /d/-final words (e.g., *feed*) was initially less accurate than recognition of /t/-final words (e.g., *feet*). Also as predicted, recognition accuracy for /d/-final words increased steadily from 0.44 (SD = 0.19) on day 1 to 0.62 (SD = 0.20) on the final day in week 4 in the L2-accented exposure group, and from 0.41 (SD = 0.21) to 0.68 (SD = 0.22) in the L1-accented exposure group.

We fit a mixed-effect logistic regression (Jaeger, 2008) to the test data using the *lme4* package in R (Bates et al., 2015). The analysis predicted accuracy (1 = correct, 0 = incorrect) from the full factorial of exposure condition (effect-coded, -0.5 = L1-accented exposure vs. +0.5 = L2-accented exposure), category (effect-coded, -0.5 = /t/- vs. +0.5 = /d/-final words), and test block (1–15 as a numeric variable, scaled by dividing by two standard deviations, Gelman, 2008). Coding test block as a numeric variable allowed us to examine whether incremental, repeated exposure resulted in cumulative improvement in the test performance. We also report in the Supplementary material on a separate analysis where we coded test block as an ordered categorical variable. We began with the maximal random effect structure justified by the design and stepwise removed higher-order interactions in the event of convergence failure. The final model included random by-participant intercepts and slopes for category, as well as by-item intercepts and slopes for exposure condition, category, and their interaction.

Both groups’ overall performance improved significantly across time, as suggested by a significant main effect of test block ( $\hat{\beta }$ = 0.35, SE = 0.05, *z* = 6.59, *p* < 0.0001). The test block-by-category interaction was also significant ( $\hat{\beta }$ = 0.10, SE = 0.11, *z* = 9.53, *p* < 0.0001), indicating that the improvement differed between /d/- and /t/-final words. A follow-up simple effects analysis found recognition accuracy significantly increased for /d/-final words ( $\hat{\beta }$ = 0.85, SE = 0.07, *z* = 11.79, *p* < 0.0001) and significantly *de*creased for /t/-final words ( $\hat{\beta }$ = −0.16, SE = 0.08, *z* = −2.01, *p* < 0.05). The overall improvement across test blocks is thus driven by the larger improvements for /d/-final words than the decreased accuracy for /t/-final words (0.85 vs. –0.16 log-odds).

To our surprise, and in contrast to an earlier, single-session experiment (Xie et al., 2017), no advantage of L2-accented exposure was observed. Neither the main effect of exposure ( $\hat{\beta }$ = −0.01, SE = 0.17, *z* = −0.07), its two-way interaction with test block ( $\hat{\beta }$ = −0.15, SE =0.10, *z* = −1.40), nor its three-way interaction with test block and category ( $\hat{\beta }$ = −0.10, SE = 0.21, *z* = −0.46) was significant. A post-hoc by-item analysis further confirmed that both groups responded similarly to each of the five minimal pairs across the five sessions (Supplementary material).

In summary, we found cumulative improvements in recognition accuracy on the L2-accented exposure and test tokens. This validates the new testing paradigm and demonstrates that it is effective for tracking long-term changes in recognition accuracy. However, contrary to our expectation, this effect did not depend on whether exposure involved L2-or L1-accented speech.

## 4 General discussion

The repeated exposure-test paradigm we explored here holds the potential to bridge two key areas of work on adaptive speech perception: (1) adaptation in the first moments of encountering an (*a priori*) unfamiliar speaker/accent and (2) longitudinal accommodation through repeated environmental exposure spanning weeks, months, and even years. While often assumed, the link between adaptive changes of perception across multiple timescales has rarely been directly tested.

Previous work has shown that exposure-induced changes in speech perception can be detected even up to 1 week after exposure (Eisner and McQueen, 2006; Witteman et al., 2015), albeit sometimes with reduced magnitude (Zheng and Samuel, 2023). While these findings speak to the longevity of the adaptive changes in speech perception from even relatively brief exposure, they leave open how *repeated* exposure affects perception. This question is not only of theoretical interest, but also helps to extend scientific knowledge to various ecologically valid scenarios of nonnative speech perception. In real life, listeners often repeatedly encounter a talker and/or an accent over days and weeks. The paradigm we have begun to develop here is meant to simulate this, allowing insights into how accent adaptation develops with repeated exposure (e.g., recurring work calls with international colleagues, listening to a nonnative course instructor).

A recent paper by Bieber and colleagues has begun to address this gap via a unique data set. In their study, eight L1 listeners responded to 750 sentences recorded by 60 different NATO officers, including 44 nonnative talkers from 13 different L1 backgrounds (Bieber et al., 2023). Listeners heard 50 sentences per block and identified multiple keywords in each sentence by clicking on written words on a tablet. Critically, they completed a total of 15 blocks in multiple sessions over five to ten days. Bieber et al. (2023) found that the greatest degree of benefit from exposure to nonnative accented speech was observed in the first block (~15% increase in accuracy). Performance continued to increase at a slower rate, but with no significant loss of accuracy between sessions (with intervals of 1–4 days between sessions). This suggests that adaptation to nonnative accents can be maintained, and it accumulates over time. However, the small sample size (eight listeners) and the relatively short duration (up to 10 days) leave open the question of how adaptation may proceed when the subject pool is more heterogeneous, and exposure is more widely spaced.

Encouragingly, our findings demonstrated the feasibility of longitudinal studies with larger participant groups tested on an online platform. Over 3 weeks, we found that sustainable subject retention is possible: After the initial technical issues we encountered (avoidable in future applications of the paradigm), subject retention was high. The majority of participants who committed to the first two sessions completed all five sessions. Moreover, while the behavioral improvements continued over time, the recognition accuracy after the fifth session was still far from ceiling. This pattern of results suggests the potential for using this paradigm to explore longer-term adaptive changes in perception, possibly over months. Importantly, the current study is one of the first to examine long-term changes in the recognition of specific phonetic categories, beyond general improvements in accented speech recognition (e.g., Bieber et al., 2023). This opens avenues for research on listeners’ adaptation to underlying phonetic category representations as a driver of longitudinal perceptual change.

However, the failure to replicate the advantage of L2-accented exposure found in previous single-session experiments also points to a challenge for similar future studies. We highlight three possible reasons for this unexpected result.

First, it is possible that it was a Type II error. One question is thus whether the present experiment was under-powered compared to Xie et al. (2017). On the one hand, each of our tests employed substantially fewer test tokens compared to the original single-session study (five pairs instead of 60). On the other hand, the simulation-based stimuli selection (Section 2.2.) countered this loss of power, and the present experiment employed substantially *more* test blocks (15 instead of one). A look at participant numbers is similarly uninformative: while the unexpected loss of participants after Session 1 reduced the participants available for analysis, it still left us with more participants than the original study (70 web-based vs. 48 lab-based). None of these considerations thus point to a clear power disadvantage of the present study. Still, to empirically address this question, we conducted a power simulation (for details, see Supplementary material). These simulations indeed estimated over 95% power to detect an advantage of L2-accented exposure with the effect sizes from the original study.

A second possible explanation for the failure to detect an advantage of L2-accented exposure concerns differences in the design. Specifically, the “control” condition in a related work (Eisner et al., 2013; Xie et al., 2017) typically employed L2-accented speech without a critical /d/-final word. In contrast, the current control condition used L1-accented speech with /d/-final word present. This design may have helped the L1-accented exposure group directly contrast the L1 and L2 accents and isolate the critical phonetic differences (e.g., Cooper and Bradlow, 2016). Under this explanation, participants in the two exposure groups both improved their L2-accent recognition but for different reasons. A follow-up experiment to address this possibility is currently underway (Kurumada and Xie, in prep).

Another, mutually compatible, possibility is that the test tokens alone may have been sufficient to support adaptation. That is, participants in the L1-accented exposure group adapted to the accent-specific features through the minimal /d/-/t/ pairs heard during the test. Even though these items were unlabeled, the underlying acoustic features relevant to /d/ vs. /t/ recognition show a natural bimodal distribution along the burst dimension (Figure 2). Learning from bimodal unlabeled input has been found in previous work, though those studies involved many more tokens (e.g., Maye et al., 2002; Clayards et al., 2008; Kleinschmidt and Jaeger, 2015; Theodore and Monto, 2019). Additionally, the repeated encounter to the same set of minimal pairs could have endorsed some response strategies. It is therefore important to avoid minimal pairs or select a greater variety of test tokens (while keeping each test block short) to avoid repetition or anchoring of test tokens within and across test blocks.

In summary, we presented a new repeated exposure-test paradigm to investigate the adaptive speech perception over three weeks. The current results and the information derived from the current work lay the empirical ground for future related lines of inquiry. For interested readers, we have provided in the Supplementary material insights we gained about the administration of a longitudinal study using an online testing platform.

## Data availability statement

The datasets presented in this study can be found in online repositories. The names of the repository/repositories and accession number(s) can be found in the article/Supplementary material.

## Ethics statement

The studies involving humans were approved by Institutional Review Board University of California Irvine. The studies were conducted in accordance with the local legislation and institutional requirements. The participants provided their written informed consent to participate in this study.

## Author contributions

XX: Conceptualization, Data curation, Formal analysis, Funding acquisition, Investigation, Methodology, Project administration, Resources, Software, Supervision, Validation, Visualization, Writing – original draft, Writing – review & editing. CK: Conceptualization, Data curation, Formal analysis, Funding acquisition, Investigation, Methodology, Project administration, Resources, Software, Supervision, Validation, Visualization, Writing – original draft, Writing – review & editing.

## Footnotes

^1^https://www.prolific.co/

^2^https://www.findingfive.com/
