# Supplementary material for: From first encounters to longitudinal exposure: a repeated exposure-test paradigm for monitoring speech adaptation
Source: Front Psychol. 2024 May 30;15:1383904. doi: 10.3389/fpsyg.2024.1383904 (PMC11169900; doi:10.3389/fpsyg.2024.1383904)
Supplement: Supplementary file 1 [file Data_Sheet_1.pdf]

Xie, X., & Kurumada, C. (2024). From first encounters to longitudinal exposure: a repeated exposure-test paradigm for monitoring speech adaptation. *Front. Psychol. 15*:1383904. doi: 10.3389/fpsyg.2024.1383904

Supplementary Material

1 Subject Demographics

On the online participant recruitment platform, Prolific, we screened participants for the following inclusion criteria: (a) 18-45 years of age, (b) native English proficiency, (c) monolingual upbringing, and (d) current residence within the 50 states of the United States. At the end of the first session (Day 1), we administered a language background questionnaire to collect information about their prior experience and familiarity with Mandarin-accented speech. Across age groups, we observed a decrease in attrition rate as participants' age increased: 61% for ages 18-24, 24% for ages 25-34, and 23% for ages 35-45. No clear pattern of attrition was observed depending on the geographic location of participants by state.

2 Recruitment and Retention

Recruitment and retention of participants is a notorious challenge for longitudinal studies. Below, we summarize the steps we took to minimize accidental data loss and to facilitate continued participation in the five sessions. Despite our best efforts, we did encounter a technical issue, resulting in a loss of 30 participants (23% of the 127 participants originally recruited). This issue was resolved since the second session and has not occurred since. Below we report a number of procedures that may help promote retention. We hope that this information will be useful to researchers interested in conducting multi-day online experiments.

Clear and continuous messaging and expectation management: In the initial recruitment, we listed all the dates of the five sessions and clearly stated that eligible and interested participants must take part in all of the sessions. This message was repeated at the end of each session. Additionally, we used Prolific's messaging function to send out a thank-you message following each session and a reminder message the day before the upcoming session. This messaging function was particularly useful to receive comments and feedback from participants as well. For increased ease of scheduling, we opened the experiment at a fixed time (e.g., 9am Pacific Time) throughout the five sessions.

Payment: Each participant received a base payment of \$2.50 after each session with a completion bonus of \$2 after the final session. To boost engagement, an additional \$0.50 bonus/session was given to those participants who achieved the average accuracy of 80% or more during the exposure phase. The bonus payments were promised at the beginning of the initial session, and the information was repeated in the reminder emails.

Technical difficulty: We encountered only one technical issue, which affected participants in the second session. We had announced that the study would open at 9am Pacific time, some of the subjects came back and tried to access the experiment before this time. While some of those did participate in the study at a later time on the scheduled day, others did not. This resulted in the largest proportion of our subject attrition (23.6%, 30/127 participants). To prevent this from happening, we added additional emphasis in our reminder email about the study start time for the subsequent sessions.

Attrition rates over five sessions: Once the initial problem was resolved, we were able to achieve a successful retention rate for the subsequent sessions. In fact, the rate of participant attrition dropped significantly as the sessions progressed (Supplementary Figure 1).

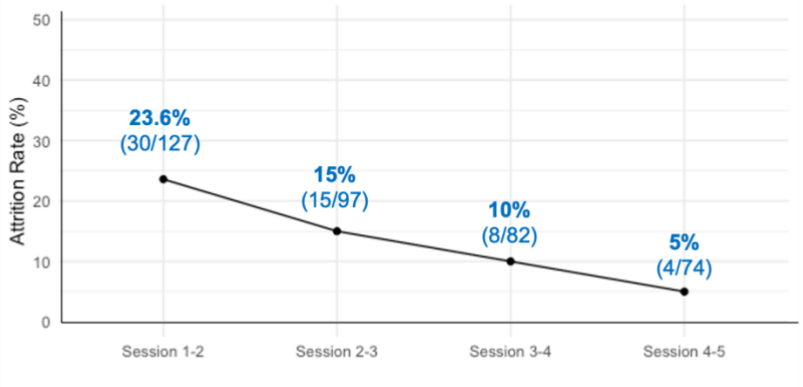

Supplementary Figure 1. Participant attribution rates across the 5 sessions.

Our impression was that after a few sessions, participants were committed to completing the five sessions. Apart from inevitable scheduling conflicts and accidental no-shows, participants were generally cooperative. For a study with a longer duration or larger time interval (e.g., monthly assessments), it may be advisable to increase the bonus payment amount and the frequency of the reminders.

3. Main analysis after excluding participants based on Mandarin-accent exposure

As described in the main text, four out of 70 participants reported having regular exposure to Mandarin Chinese and/or Mandarin-accented English from a family member or a close friend. To examine whether the inclusion of these participants significantly affected the results, we conducted the same GLMM analysis reported in the main text after excluding these four participants.

All the effects we reported in the main text remained significant. Specifically, both groups' overall performance steadily improved across time, as suggested by a significant main effect of test block ($\hat{\boldsymbol{\beta}}$ = .34, SE = .05, z = 6.27, p < .0001). The test block-by-category interaction was also significant ($\hat{\boldsymbol{\beta}}$ = .10, SE = .10, z = 9.40, p < .0001), indicating that the improvement was greater for the /d/-final words than the /t/-final words. Crucially, replicating the main analysis reported in the main text, neither the main effect of exposure ($\hat{\boldsymbol{\beta}}$ = .01, SE = .17, z = .08), its two-way interaction with test block ($\hat{\boldsymbol{\beta}}$ = -.09, SE =.11, z = -.86), nor three-way interaction between condition, test block and category ($\hat{\boldsymbol{\beta}}$ = .-.02, SE = .22, z = -.09, p = .93) was significant. Thus, we conclude that the current results are not driven by the small proportion (4%) of the participants who reported close contact with Mandarin-accented talkers.

4. Sliding-difference coding

Test block (e.g., blocks 1-3 in Session 1) as an independent variable can be encoded in a GLMM model in at least two different ways. One is as a numerical variable, as we report on in the main text, and the other is as an ordered categorical variable. The former allows us to test our core hypothesis: increased exposure to L2-accented speech will facilitate L1 listeners' recognition of the initially

difficult category (e.g., a word-final /d/ sound in Mandarin-accented English). However, this may overlook more subtle effects of individual test blocks. For example, listeners' performance may change only during the first few test blocks and plateau in a later block. The numerical coding of test blocks would not allow us to identify such non-linear changes in performance.

To address this issue, we conducted an additional analysis of the data using sliding-difference coding of the test block (Schad et al., 2020). Sliding difference coding compares levels of a categorical variable to a reference level by calculating the differences between each level and the previous level. Here, the coefficients estimated for each level in a generalized linear mixed effects model (GLMM) represent the difference between that level (e.g., test block 2) and the previous level (e.g., test block 1). Below, we report these additional analyses that were performed on the results reported in the main text. The details of the models and model results can be found in the project's OSF entry osf.io/5xfpe/.

The mixed-effect logistic regression model was identical to what we reported in Section 2.4 except for the coding of the test block. The analysis predicted accuracy (1 = correct, 0 = incorrect) from the full factorial of exposure condition (contrast sum-coded, -.5 = control vs. +.5 = target), and category (contrast sum-coded, -.5 = /t/- vs. +.5 = /d/-final words). The 15 test blocks over five days were sliding-difference coded as an ordered categorical variable (block 1-15, 14 levels of comparison such as block 2 vs. 1, 3 vs. 2). We began with the maximal random effect structure justified by the design and removed higher-order interactions in the event of model convergence failure. The final model included random by-subject intercepts and slopes for category as well as by-item intercepts and slopes for exposure condition, category, and their interaction.

Of the 14 levels of test block, 10 were significant with positive coefficients. This means that the recognition improved steadily with increasing exposure, regardless of the exposure condition. 10 /14 interaction terms between category and test block had positive coefficients, indicating that listeners consistently improved their recognition of /d/-final words to a greater extent than /t/-final words, although none of them reach statistical significance. No interaction term between exposure condition and test block approached significance, nor did a three-way interaction term between exposure condition, category, and test block. Similar to what was reported in the main text, the effect of condition was not a significant predictor of listeners' recognition accuracy.

In summary, the GLMM model with the categorical variable of test block revealed that the L1 listeners consistently improved their performances with the increased exposure. This supports the conclusion we drew from the main analysis that adaptive changes in the recognition of L2-accented speech are cumulative. They are *not* reset between instances of exposure and continue with as the input accumulates over time. As in the main analysis, there was no significant effect of exposure condition.

Schad, D. J., Vasishth, S., Hohenstein, S., & Kliegl, R. (2020). How to capitalize on a priori contrasts in linear (mixed) models: A tutorial. *Journal of Memory and Language, 110.* *https://doi.org/10.1016/j.jml.2019.104038*.

5. By-item analysis

We fit another GLMM to the test data predicting accuracy (1 = correct, 0 = incorrect) using the following fixed-effects factors: exposure condition (contrast sum-coded, -.5 = control vs. +.5 =

target), category (contrast sum-coded, -.5 = /t/- vs. +.5 = /d/-final words), **test block (1-15 as a numeric variable, scaled by dividing by two standard deviations**), and item pair (treatment-coded for the five minimal pairs). To examine whether the two exposure groups responded similarly to the specific test items, we conducted post-hoc simple effects analysis. Two major results are noteworthy from this analysis: first, at each level of the item, there was significant effect of category and block, as well as category-by-block interactions, suggesting strong item effects overall. Second, and more important to our current question, there was no interaction between condition and the other two factors (block and category) for any of the items. This suggests that the two groups responded similarly to the five minimal pairs.

	Estimate	Std.Error	z value	Pr(>|z|)	
(Intercept)	1.25	0.09	13.24	< 2e-16	***
Item (fried/flight)	-1.13	0.09	-12.01	< 2e-16	***
Item (kid/kit)	-0.44	0.08	-5.57	0.000	***
Item (plod/plot)	-0.23	0.08	-3.01	0.003	**
Item (wed/wet)	1.18	0.14	8.20	0.000	***
Item (feed/feet):Category	0.54	0.22	2.47	0.013	*
Item (fried):Category	-4.70	0.24	-19.74	< 2e-16	***
Item (kid):Category	1.97	0.22	9.09	< 2e-16	***
Item (plod):Category	-1.34	0.22	-6.21	0.000	***
Item (wed):Category	-3.82	0.32	-11.79	< 2e-16	***
Item (feed):Block	0.24	0.11	2.19	0.029	*
Item (fried):Block	0.17	0.14	1.16	0.245	
Item (kid):Block	0.31	0.11	2.76	0.006	**
Item (plod):Block	0.38	0.11	3.50	0.000	***
Item (wed):Block	0.66	0.26	2.52	0.012	*
Item (feed):Condition	-0.03	0.19	-0.17	0.866	
Item (fried):Condition	-0.22	0.21	-1.02	0.307	
Item (kid):Condition	0.15	0.19	0.79	0.430	
Item (plod):Condition	-0.11	0.19	-0.57	0.566	
Item (wed):Condition	0.35	0.31	1.16	0.247	
Item (feed):Category:Block	1.51	0.22	6.77	0.000	***
Item (fried):Category:Block	0.23	0.29	0.78	0.434	
Item (kid):Category:Block	0.72	0.22	3.20	0.001	**
Item (plod):Category:Block	0.73	0.22	3.34	0.001	***
Item (wed):Category:Block	1.39	0.53	2.63	0.009	**
Item (feed):Category:Condition	0.10	0.43	0.23	0.818	
Item (fried):Category:Condition	-0.69	0.47	-1.45	0.146	
Item (kid):Category:Condition	0.16	0.43	0.36	0.719	
Item (plod):Category:Condition	-0.82	0.43	-1.90	0.057	.
Item (wed):Category:Condition	-1.94	0.65	-3.00	0.003	**
Item (feed):Block:Condition	-0.10	0.22	-0.45	0.657	
Item (fried):Block:Condition	0.12	0.29	0.43	0.670	

Item (kid):Block:Condition	-0.09	0.22	-0.39	0.698	
Item (plod):Block:Condition	-0.45	0.22	-2.08	0.037	*
Item (wed):Block:Condition	0.11	0.53	0.21	0.836	
Item (feed):Category:Block:Condition	-0.24	0.44	-0.55	0.586	
Item (fried):Category:Block:Condition	0.42	0.58	0.73	0.467	
Item (kid):Category:Block:Condition	0.16	0.45	0.36	0.716	
Item (plod):Category:Block:Condition	-0.54	0.43	-1.25	0.212	
Item (wed):Category:Block:Condition	-0.65	1.05	-0.62	0.535	

6. Post-hoc power simulations using data from Xie et al (2017)

One possible reason for the current failure to replicate the single-session study by Xie et al. (2017) is a loss of statistical power due to the administrative error after the first session. To address this possibility, we conducted a *post hoc* power analysis using the Xie et al. data. Specifically, we asked how much power the current repeated-exposure-test design would have *if the effect size were 100%, 80%, 50% etc. of Xie et al.* To do this, we took the following two steps.

First, we reanalyzed the data from Xie et al. using the same GLMM as reported for the current study. The analysis predicted accuracy (1 = correct, 0 = incorrect) from the full factorial of exposure condition (effect-coded, -.5 = Control (i.e., no /d/ exposure) vs. +.5 = Target (i.e., with /d/ exposure)), category (effect-coded, -.5 = /t/- vs. +.5 = /d/-final words), and an interaction term between the two. This equated the results of the two models in terms of the methods of analysis. The following summarizes the fixed effects for the Xie et al. data:

	Estimate	Std.Error	Z	Pr(>|z|)
(Intercept)	1.0166	0.1227	8.288	<2e-16 ***
Condition (with /d/ exposure vs. without)	0.1850	0.1492	1.240	0.2151
Category (/d/ vs. /t/)	-0.1782	0.3931	-0.453	0.6503
Condition: Category	0.9526	0.3973	2.398	0.0165 *

This supports a significant interaction between condition and category, suggesting that the L2-accented exposure resulted in a greater amount of benefit for the recognition of the /d/-category than the L1-accented exposure.

Second, we conducted power simulations to estimate the power of the current five-day repeated exposure-test experimental design based on the effect sizes derived in the mixed-effect model above. We used the package Mixedpower (Kumle et a., 2021) to simulate replications of our experiment. For these simulations, we set all fixed and random effects to the ones reported in the main text, with three exceptions. First, we set the effect of condition on Test 1 to zero i.e., We assume no difference between the two conditions (L1-accent exposure vs. L2-accent exposure) prior to exposure. Second, we set two fixed effects that jointly describe the effect of exposure to values based on Xie et al. Specifically, we set the test block × condition interaction to be a multiple (*d*) of the estimate observed for the condition effect in Xie et al., and we set the test block × condition × category interaction to be a multiple (again *d*) of the estimate observed for the condition × category interaction in Xie et al. This allowed us to simulate the cumulative effects of exposure in our experiment, assuming that each exposure token in our experiment had *d*-times the effect observed in Xie et al.

We considered 6 different values for *d*, ranging from 0 to 1. For a value of 0, exposure has no effect, and so the L1- and L2-accent exposure conditions have identical accuracy after the fifth exposure session (on Test Block 15). For a value of 1, the expected effect after the fifth exposure is five times that observed by Xie et al. For each of the six values for *d*, we simulated 201 instances of our experiment with 70 participants, resulting in 1206 simulated data sets and 1206 analyses thereof, which are summarized in Figure S2 (for details, see R code at osf.io/5xfpe/).

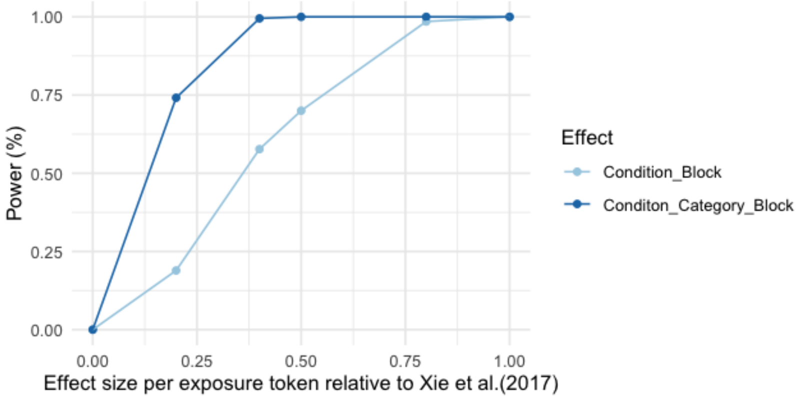

Supplementary Figure 2. Estimated statistical power of the current experimental design based on the effect sizes derived from Xie et al.'s (2017) GLMM model. For ease of comparison, the x-axis shows the effect size per exposure token relative to Xie et al. 's. "0" denotes a scenario where the exposure has no effect on listeners' recognition. "1" denotes a scenario in which each exposure token in the current study changed listeners' recognition of the test items to the same extent as in the original Xie et al. study.

Figure 2 summarizes the results of the power simulations. The results suggest that if the effect size is approximately the same as in Xie et al., the estimated power to detect (1) the test block-by-condition-by-block interaction and (2) the test block-by-condition-by-category interaction is both close to 100% after five sessions (i.e., after 10 exposure blocks, each half the size of the exposure blocks in the original study). Even if the effect size was 50% of that in the original study, we should have 100% power to detect the condition-by-category-by-block interaction if there was a true effect. Taken together, the current power simulations suggest that the failure to replicate Xie et al. in the current study is unlikely to be due to a Type II error.

We note, however, that this conclusion should be interpreted with caution. Xie et al. and the current experiment differed in some experimental variables. For example, the two conditions in Xie et al. both used L2-accented speech, with vs. without /d/ final words. On the other hand, the two conditions in the current study used L2- vs. L1-accented speech, both with /d/-final words. In addition, as we discussed in the General Discussion, the repeated-exposure-test paradigm may have encouraged participants to rely on some response strategies (e.g., response anchoring), which were less feasible in Xie et al. Precise power estimates need to be made after these design variables are equated, which we are now well equipped to do for our future studies.

Kumle, L., Võ, M. L., & Draschkow, D. (2021). Estimating power in (generalized) linear mixed models: an open introduction and tutorial in R. *Behav Res*. doi:10.3758/s13428-021-01546-0
